# Mobile Mammography in Underserved Populations: Analysis of Outcomes of 3,923 Women

**DOI:** 10.1007/s10900-013-9696-7

**Published:** 2013-05-15

**Authors:** Sandra E. Brooks, Tina M. Hembree, Brent J. Shelton, Sydney C. Beache, Greta Aschbacher, Philip H. Schervish, Mark B. Dignan

**Affiliations:** 1 Centers for Prevention and Wellness, Norton Healthcare, Louisville, KY USA; 2 Division of Cancer Biostatistics, College of Public Health, Markey Cancer Center, University of Kentucky, Lexington, KY USA; 3 Centers for Prevention and Wellness, Norton Healthcare, 3999 Dutchmans Lane, Suite 3C, Louisville, KY 40207 USA; 4 Prevention Research Center, Internal Medicine, Markey Cancer Center, University of Kentucky, Lexington, KY USA

**Keywords:** Breast cancer, Mobile mammography, Underserved populations, Outcomes, Disparities

## Abstract

Mobile health units are increasingly utilized to address barriers to mammography screening. Despite the existence of mobile mammography outreach throughout the US, there is a paucity of data describing the populations served by mobile units and the ability of these programs to reach underserved populations, address disparities, and report on outcomes of screening performance. To evaluate the association of variables associated with outcomes for women undergoing breast cancer screening and clinical evaluation on a mobile unit. Retrospective analysis of women undergoing mammography screening during the period 2008–2010. Logistic regression was fitted using generalized estimating equations to account for potential repeat annual visits to the mobile unit. In total, 4,543 mammograms and/or clinical breast exams were conducted on 3,923 women with a mean age of 54.6, 29 % of whom had either never been screened or had not had a screening in 5 years. Age < 50 years, lack of insurance, Hispanic ethnicity, current smoking, or having a family relative (<50 years of age) with a diagnosis of cancer were associated with increased odds of a suspicious mammogram finding (BIRADS 4,5,6). Thirty-one breast cancers were detected. The mobile outreach initiative successfully engaged many women who had not had a recent mammogram. Lack of insurance and current smoking were modifiable variables associated with abnormal screens requiring follow up.

## Background

Many studies indicate that breast cancer health disparities exist among women of different races, ethnicities, socioeconomic statuses, geographic locations and age [1, 2]. According to the Centers for Disease Control and Prevention (CDC), National Program of Cancer Registries (NPCR) while the age-adjusted incidence rate of breast cancer among black females is slightly lower than that of white females, the mortality rates from breast cancer are significantly higher among black females [3, 4]. Rates of mammography screening for breast cancer, tend to be lower among women who are not insured, have a minimal amount of formal education, and are of a non-white race [5–7]. Inadequate screening and high breast cancer mortality are of concern in Kentucky as this state has one of the highest breast cancer mortality rates in the US [8]. In addition, a 2006 report, Smigal et al. [7] determined that 40–50 % of women with a diagnosis of breast cancer had not had a mammogram within the past year and the mortality rate of Black women was 37 % higher than that of White women.

Mobile health units are increasingly utilized to address barriers to mammography screening [9]. Despite the existence of mobile mammography outreach throughout the US, there is a paucity of data describing the populations served by mobile units and the ability to reach underserved populations and address disparities [10–15]. To explore this issue, we sought to evaluate the demographics of the women screened on a mobile unit as part of a prevention program in Louisville, KY. We additionally evaluated screening outcomes and variables associated with the need for additional follow up in order to assess the ability of the prevention program to address health disparities and identify women with breast cancer. The prevention program focuses its efforts in high cancer incidence and high mortality areas in Jefferson County defined as high risk areas. The program utilizes an approach involving community health outreach workers working with community partners in the program defined high risk areas to identify sites for screening. Screenings are conducted on a 40 foot mobile unit equipped with digital mammography and exam room. The mobile unit team includes an advanced practice nurse or physician, registered nurses, community health workers and technical support staff. Each eligible woman (40–75, no screening within the past year) is provided educational counseling, a focused history and physical examination and screening mammogram or referral for a diagnostic mammogram if indicated. Women who were not insured at the time of screening were invited to join a program that would underwrite the cost of payment for screening and follow up. Post screening, prevention program nurse navigators and physicians reviewed all results and made appropriate referrals for diagnostic follow up or specialty services for all suspected cancers.

## Methods

We conducted a retrospective review of screening and follow up data obtained for women screened on the mobile mammography unit in Jefferson County, KY during the period 2008 to 2010 as part of the prevention program in Louisville Kentucky. The study population consisted of 3,923 women undergoing 4,543 screening mammograms and/or clinical breast exams on a mobile unit.

The radiologist reviewing the studies scored each mammogram using the BIRAD system of coding 0–6. BIRAD 0 scores were considered incomplete and required additional follow up. BIRAD Scores of 1–3 were considered normal, benign, or probably benign. Mammograms coded as BIRADS 4-6 were considered suspicious or malignant and required immediate referral to a diagnostic mammogram or specialty physician (Table 1, 2).

**Table 1:** BIRADS classification

Category	Diagnosis	Number of criteria
0	Incomplete	Your mammogram or ultrasound didn’t give the radiologist enough information to make a clear diagnosis; follow-up imaging is necessary
1	Negative	There is nothing to comment on; routine screening recommended
2	Benign	A definite benign finding; routine screening recommended
3	Probably benign	Findings that have a high probability of being benign (>98 %); six-month short interval follow-up
4	Suspicious abnormality	Not characteristic of breast cancer, but reasonable probability of being malignant (3–94 %); biopsy should be considered
5	Highly suspicious of malignancy	Lesion that has a high probability of being malignant (≥95 %); take appropriate action
6	Known biopsy proven malignancy	Lesions known to be malignant that are being imaged prior to definitive treatment; assure that treatment is completed

D’Orsi et al. [29]

**Table 2:** Variable trends over the 3 year study period (2008, 2009, 2010)

Live in high risk area		Age		CBE abnormality		Current smoker		Recency of screening		Race		Mammographic abnormality		Personal history of cancer	
Yes	2,349(52 %)	<50	1,533(34 %)	Yes	158(3 %)	Yes	1,343(30 %)	Never	729(16 %)	Black	2,217(45 %)	BIRAD 4,5,6	188(4 %)	Yes	300(7 %)
No	2,192(48 %)	50+	3,008(66 %)	No	4,358(96 %)	No	3,162(70 %)	Within last 5 yrs	3,104(68 %)	White	2,496(50 %)	BIRAD 1,2,3	3,991(88 %)	No	4,167(92 %)
Missing	1(< 1 %)	Missing	1(< 1 %)	Refused/not done	27(1 %)	Missing	38(1 %)	More than 5 yrs	613(13 %)	Other	227(5 %)	BIRAD 0	236(5 %)	Missing	67(1 %)
								Missing	97(2 %)	Missing		Missing	128(3 %)		

Have a PCP?		Ethnicity		Breast cancer		Family history of cancer (with age <50 years)		Insurance status	
Yes	3,229(71 %)	Hispanic	513(11 %)	Yes	31(1 %)	Yes	1,415(31 %)	None	2,583(57 %)
No	1,122(25 %)	Non-Hispanic	3,955(87 %)	No	4,512(99 %)	No	3,052(67 %)	Medicaid	929(20 %)
Missing	192(4 %)	Other	75(2 %)			Missing	76(2 %)	Medicare	368(8 %)
								Private	599(13 %)
								Missing	64(1 %)

Variable n = 4,543

Given the potential impact on the program in navigating individuals for follow up, detailed analysis was performed on all tests requiring additional follow up (BIRAD 0 and BIRADs 4–6).We performed logistic regression to evaluate variables (age, race, ethnicity, insurance, smoking status, family or personal history of cancer) associated with BIRAD 0 (incomplete) or BIRAD 4–6 (suspicious or malignant) (see Table 3).

**Table 3:** Multi variable logistic regression

Risk factor		BIRAD 4.5.6 Mammography (compared to normal)			BIRAD 0 Mammography (compared to normal BIRAD 1,2,3)		
		BIRAD 4–6 versus 1–3			BIRAD 0 versus 1–3		
		Odds ratio	95 % CI	*p*	Odds ratio	95 % CI	*p*
High risk area	Low versus high	0.78	(0.55, 1.11)	0.16	1.19	(0.86, 1.64)	0.29
Age	<50 versus ≥50	1.65	(1.17, 2.31)	<0.01	1.25	(0.91, 1.72)	0.16
Screening recency	(Within 5 years versus never or beyond 5 years)	0.90	(0.65, 1.26)	0.56	0.64	(0.47, 0.89)	<0.01
Race	(B versus W)	0.83	(0.57, 1.20)	0.32	0.68	(0.48, 0.96)	0.03
	(B versus oth)	1.06	(0.45, 2.52)	0.89	0.51	(0.27, 0.98)	0.04
	(W versus oth)	1.28	(0.56, 2.93)	0.56	0.76	(0.40, 1.41)	0.38
Primary care physician	(N versus Y)	1.22	(0.84, 1.78)	0.29	1.50	(1.08, 2.09)	0.02
Insurance status	None versus private	1.63	(1.04, 2.55)	0.03	0.87	(0.62, 1.22)	0.42
Ethnicity	(H versus NH)	1.87	(1.17, 2.98)	<0.01	0.81	(0.50, 1.31)	0.39
Current smoking history	(N versus Y)	0.65	(0.46, 0.90)	0.01	0.86	(0.62, 1.19)	0.36
Personal Hx w cancer	(N versus Y)	0.69	(0.38, 1.25)	0.22	0.96	(0.53, 1.74)	0.90
Family Hx w cancer (age <50)	(N versus Y)	0.64	(0.47, 0.88)	<0.01	1.07	(0.77, 1.47)	0.70

**Fig. 1:**
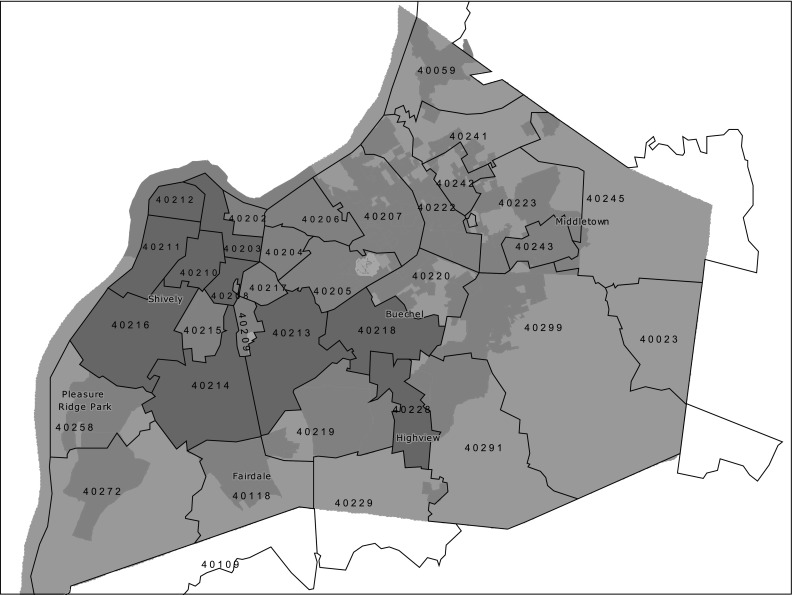
High risk areas zip codes in Jefferson County/Louisville-Metro, KY

Given that zip code of residence is associated with economic and health indicators, we defined a high risk area as residence in a zip code categorized by high cancer mortality and high poverty in Jefferson County based on population and cancer statistics provided by the Kentucky Cancer Registry, the US 2000 Census and the Louisville Metro Health Equity report [16]. The high risk area zip codes defined for this study were 40,203, 40,210, 40,211, 40,212, 40,215, 40,216, 40,218, 40,219, and 40,228 (see Fig. 1).

Logistic regression fitted in the context of generalized estimating equations (GEE) was performed to fit the set of risk factors to the primary breast abnormality binary outcome (normal vs. abnormal). GEE is a well-known and well-documented approach to account for the repeated visits by some of the study participants over the 3 year study period. In addition to logistic modeling for the two primary outcomes, two additional logistic regression models were fit to assess whether risk factors were associated with incomplete mammographic results compared to normal and abnormal results. The final models reported included all of the risk factors considered (a main effects model with all a priori main effects included in the final model). The goal of this analysis was to explore associations between abnormality outcome and all 10 covariates simultaneously. To this end, the odds ratios corresponding to each of the 10 main effects from the multivariable model were reported as they are adjusted for the effects of remaining covariates in the model (this also increases precision). The analysis additionally reflected modeling of presence of breast abnormality over potential repeat annual visits. (Table 3). Nine of the ten covariates considered were binary in nature with race being coded as “black”, “white”, and “other”. The reference cell for race in the multivariable model was the “white” category. Results for race presented in Table 3 include all 3 pairwise comparisons for race. These were accomplished using appropriate contrasts applied to the overall multivariable model.

To determine if racial disparities in stage distribution existed for the women diagnosed with cancer during the study period, Fisher’s Exact test was used to compare stage of breast cancer distributions between blacks and whites. (Table 4).

**Table 4:** Breast cancer stage distribution by race/ethnicity

	African American (45.2 %)	White (54.8 %)	Hispanic (9.7 %)	Non- Hispanic (90.3 %)
Stage 0	3 (9.7 %)	6 (19.3 %)	0	9 (29.0 %)
Stage I	2 (6.4 %)	8 (25.8 %)	1 (3.2 %)	9 (29.0 %)
Stage II	5 (16.1 %)	1 (3.2 %)	1 (3.2 %)	5 (16.1 %)
Stage III	2 (6.4 %)	1 (3.2 %)	0	3 (9.7 %)
Stage IV	1 (3.2 %)	1 (3.2 %)	1 (3.2 %)	1 (3.2 %)
Unknown	1 (3.2 %)	0	0	1 (3.2 %)

n = 31

## Results

### Screening Population

Descriptive statistics for the women undergoing mammography screening on the mobile unit for the three year period are displayed in Table 2. Eleven percent of women were Hispanic and 48 % were African American, 43 %White and 5 % other. At the time of screening, 29 % of the women had either never had a mammogram or had not had one in five years or more. During the study period, 52 % of women resided in high risk areas. Fifty-six percent of women screened lacked health insurance and 25 % of women did not have a primary care physician.

### Variables Associated with Abnormal or Incomplete Results

The following variables were associated with a mammogram that was suspicious or consistent with malignancy (coded as BIRAD 4-6) or a clinical examination that required immediate referral for a diagnostic mammogram: Age under 50, Hispanic ethnicity, absence of insurance, current smoking history, and the existence of a relative less than 50 years of age with cancer. (Table 2).

To evaluate additional variables associated with results requiring follow up, we compared mammograms that were associated with BIRADS 0 (incomplete) to those with normal results (Table 3). Women who had not been screened within last 5 years, Caucasian women, women of Hispanic ethnicity and women without a primary care physician had higher odds of having a mammogram coded as incomplete BIRADS 0, resulting in the need for additional screening compared to nonwhite or non-Hispanic women, those women screened in the past 5 years or who had a primary care physician.

All of the women with suspicious mammograms were followed up to diagnostic resolution. A total of 31 women were diagnosed with breast cancer during the time period, representing 0.79 % (31/3,923) of the total screening population compared to an age adjusted rate of 0.122 % in the general population. (http://apps.nccd.cdc.gov/uscs/toptencancers.aspx date accessed 10-1-12). The stage distribution of women diagnosed with cancer is found in Table 3. The mean age of women diagnosed with cancer was 55 years (SD = 9.53). Black/African American women were more likely to be diagnosed with Stage II-IV disease compared to white women (61 vs. 18 % *p* = 0.0355, two-sided Fisher’s Exact test). Of the 31 women with breast cancer, 21 (68 %) had no insurance, 6 (19 %) were insured privately, 2 (6 %) were insured by Medicare and 2 (6 %) by Medicaid. Twenty-nine percent had either never been screened or had not been screened in the past 5 years. Eleven women (35 %) reported not having a primary care physician. Eighty-six percent of the black/African American women with cancer either had no insurance or were insured by Medicaid at the time of screening compared to 65 % of whites, although this was not statistically significant.

## Conclusions

Although previous studies indicate that offering on-site mammography at community-based sites where women gather is an effective method for increasing breast cancer screening rates among underserved women, there is a paucity of data evaluating the actual outcomes of those screening efforts [17]. This study is one of the first to examine variables associated with mammography screening outcomes for women receiving mammograms on a mobile unit. This study demonstrated the screening program attracted a high percentage of women who were uninsured and had not been screened in the last 5 years.

We determined that age (<50), lack of insurance, Hispanic ethnicity, current smoking, and reporting a relative diagnosed with cancer under the age of 50 were all related to a higher likelihood of requiring follow up after a screening mammogram. Women without a recent mammogram, White women, and those without a primary care physician were more likely to have a BIRAD 0 (incomplete) mammogram requiring follow up. The reasons for these associations were not evaluated by this study; however previous reports indicate an association of race and prediction of incomplete screening mammography [18]. In addition, in as much as past films are reviewed to aid in the disposition of mammography findings- views beyond initial screening may be required in those women for whom no prior screening history exists or is available- this may explain the association of screening recency and BIRAD 0 results in this study. This is an important consideration in resourcing mobile mammography outreach as it may be necessary to navigate such women to additional testing and follow up once the initial screen is complete.

The potentially modifiable variables that were associated with the need for additional follow up: lack of insurance, no prior screening, lack of a primary care physician are variables associated with healthcare practices, access to care and socio economic status. Current smoking was also found to be associated with a higher likelihood of requiring follow up. As there is insufficient evidence to support a link between breast cancer and tobacco the reasons for this finding is not clear and is deserving of further study [19].

During this time period, the Mobile health unit, traveled to more than 200 locations to conduct nearly 4,000 mammograms and increased access to preventive healthcare services in high-risk or underserved communities and populations. In addition, the community outreach workers provided encouragement and the provision of low cost/no cost services and presence of a provider on site to perform clinical breast examinations and individual counseling, resulted in the ability to identify a large percentage of women who had not been screened in five years or more. Previous studies have documented barriers to entry into the health system for low income women with suspected breast cancer, resulting in delays in diagnosis [20]. In order to address those barriers, our program linked clinical evaluation, screening with follow up and this study was successful in identifying cancers at a rate higher than would be expected in the general population.

Consistent with the established literature, we found that African American women were more likely to be diagnosed with Stage II–IV disease compared to White women in this study [21, 22]. The rate of detection of cancer in this population at 0.79 % is higher (binomial test *p* < 0.0001) than would have been expected for a general population (SEER 2004-2008 rate of 0.124 %) an indication that the team successfully identified a high risk group of women on whom to focus our efforts [23].

The percentage of Hispanic women in this study which averaged 12 % was higher than that of the general population in Louisville due to a dedicated Hispanic/Latino outreach initiative imbedded within the outreach program. The Hispanic women in this study who were largely uninsured were also at higher risk for suspicious findings on mammogram that required diagnostic follow up.

This study reaffirms that uninsured women are more likely to receive less frequent or no cancer screening, resulting in delayed diagnosis, delayed treatment, and advanced stage at the time of diagnosis. It also demonstrates the outcomes associated with programs that link screening with follow up to diagnostic resolution. Such programs are needed as it is known that the uninsured suffer from negative health consequences due to their lack of access to necessary medical care, and the cost of care when received is substantially higher [24, 25].

In a review of the 2007 health tracking Household Survey, Kullgren et al. [26] 15 % of US adults reported affordability barriers and 21 % experienced non-financial barriers that led to unmet need or delayed care. Women and those with lower incomes or with at least one chronic illness have higher adjusted prevalence of non-financial barriers. Outreach initiatives such as the one described address both financial (no cost screening, in community locations) and non-financial barriers (one on one counseling) to promote access and streamline care for those with identified abnormalities.

Commonly raised concerns about mobile mammography include quality control, cost-effectiveness, and patients’ compliance with follow-up recommendations. Mobile mammography units, including our Mobile Prevention Center, are subject to the same strict oversight and guidelines for breast screening provided by the Mammography Quality Standards Act and Program as any mammography facility. The Mobile Prevention Center is inspected annually by the Kentucky Department of Public Health as well as the American College of Radiology. Compliance was addressed through physician supervised protocols involving contacting patients by telephone, letter and certified mail [27]. All of the patients diagnosed with cancer in this study were navigated to follow up.

The limitations of our study include those associated with retrospective studies, sample bias, and the potential under reporting of suspicious mammograms due to coding. Other variables that may have been associated with BIRAD-0 or incomplete results that were not examined by this study include breast density, the body mass index of the participants. These are issues worthy of future study [10, 28].

Despite its limitations, this study is one of the largest to examine the screening outcomes of thousands of women accessing screening on a mobile health unit. Providing mobile mammography services in partnership with community organizations, can be effective in increasing access and decreasing barriers to screening hard-to-reach populations. The goal is that such efforts to identify and screen and navigate underserved women will ultimately lead to a stage shift in earlier detection of breast cancer and other chronic diseases.
